# Investigating the Contraction Pattern of the Zygomaticus Major Muscle and its Clinical Relevance: A Functional MRI Study

**DOI:** 10.1007/s00266-024-03876-8

**Published:** 2024-02-27

**Authors:** Daniel J. Rams, Michael Alfertshofer, Jakub Batko, Robert H. Gotkin, Galen Perdikis, Elżbieta Szczepanek, Andrzej Urbanik, Mateusz Koziej, Monika Ostrogórska, Sebastian Cotofana

**Affiliations:** 1 Department of Anatomy, Jagiellonian University Medical College, Kraków, Poland; 2 Department of Oromaxillofacial Surgery, Ludwig-Maximilians-University Munich, Munich, Germany; 3 Private Practice, New York, NY USA; 4 Department of Plastic Surgery, Vanderbilt University, Nashville, TN USA; 5 Department of Radiology, Jagiellonian University Medical College, Kraków, Poland; 6 Department of Dermatology, Erasmus University Medical Center, Dr. Molewaterplein 40, 3015 GD Rotterdam, The Netherlands; 7 Centre for Cutaneous Research, Blizard Institute, Queen Mary University of London, London, UK; 8 Department of Plastic and Reconstructive Surgery, Guangdong Second Provincial General Hospital, Guangzhou, Guangdong Province China

**Keywords:** Zygomaticus major muscle, Facial muscle, Contraction pattern, Facial anatomy, Muscle physiology

## Abstract

**Background:**

Our understanding of facial anatomy has significantly evolved, yet the detailed contraction patterns of facial muscles and their presentation during clinical imaging remain largely unexplored. Understanding the contraction patterns and visual presentation of these muscles, particularly the zygomaticus major could enhance pre-surgical facial assessments and the development of new treatment strategies.

**Methods:**

A total of 34 healthy young individuals (17 female, 17 male) with a mean age of 23.6 (2.4) years [range: 20–30] were investigated regarding the length, thickness, width, and angle of the zygomaticus major muscle in five different facial expressions (i.e., repose, anger, joy, surprise, and sadness) utilizing MR imaging.

**Results:**

Joyful expressions caused a reduction in muscle length to 85.6% of its original length and an increase in width (103.4%), thickness (108.4%), and facial angle (2.72°) when compared to that in repose, suggesting isotonic contraction. Conversely, expressions of anger, surprise, and sadness generally led to muscle stretching, seen through changes in length (98.9%, 104.3%, and 102.7%, respectively), width (98.8%, 96.5%, and 99.4%, respectively), and thickness (91.2%, 91.0%, and 102.7%, respectively), with variable alterations in facial angle (0.55°, 1.85°, and 1.00°, respectively) depending on the specific expression.

**Conclusion:**

This MRI-based study indicates that the zygomaticus major muscle experiences isotonic contraction, characterized by decreased length and increased width and thickness. The findings underline the importance of muscle thickness as a reliable parameter in assessing facial muscle function and offer valuable guidance for practitioners in accurately evaluating muscle performance during different facial expressions.

**No Level Assigned:**

This journal requires that authors assign a level of evidence to each submission to which Evidence-Based Medicine rankings are applicable. This excludes Review Articles, Book Reviews, and manuscripts that concern Basic Science, Animal Studies, Cadaver Studies, and Experimental Studies. For a full description of these Evidence-Based Medicine ratings, please refer to the Table of Contents or the online Instructions to Authors www.springer.com/00266.

## Introduction

Our understanding of facial anatomy has evolved rapidly during the last couple of decades and this evolution has improved our ability to apply this knowledge to both surgical and non-surgical interventions [1–7]. Utilizing surface-derived electromyography (EMG), it was recently shown by Frank et al. and by Cotofana et al. [8, 9] that facial muscles are subject to age-related changes because the measured signal of the zygomaticus major, procerus, and orbicularis oculi muscles decreased significantly in mature study participants when compared to younger controls. The authors of both studies speculated that facial muscles like skeletal muscles might be subject to a process known as sarcopenia—an age-related condition negatively affecting skeletal muscle mass and thus skeletal muscle function [8, 9]. These findings are novel and facilitate a deeper understanding of the facial aging process and the development of novel treatment strategies.

Recently, Calomeni et al. [10] found that in the largest muscle supplied by the facial nerve—the platysma—various contraction patterns exist simultaneously during its neural activation: isometric and isotonic. The spatial difference between those two contraction patterns results in the formation of platysmal banding. These muscle bands can be treated either surgically or with injectable neuromodulators [11, 12]. The novelty of that study [10] was that no detailed information had been available previously about the contraction pattern of facial muscles and whether they behave like skeletal muscles or display a different pattern due to their connection to the overlying dermis. The clinical application of such findings is the correlation between visible bands and platysmal adhesions; this helps to guide practitioners like a map to surgically or chemically release the banding phenomenon.

Facial muscles were designed to connect to the skin or other muscles and to affect various facial expressions when activated. However, facial expressions are always the result of multiple muscles being activated at the same time, i.e., multi-muscle movements, and never the result of a single muscle contracting alone [13]. During facial assessment before facial surgery such as re-animation procedures, trauma reconstruction, or face transplantation, it is important to identify which muscle is predominantly responsible for specific facial movements. Tools that can help facilitate such an assessment are either magnetic resonance imaging (MRI) or ultrasound (US) imaging; EMG is of less help due to its limited spatial resolution.

Unfortunately, it is still unknown what type of contraction pattern (isotonic vs. isometric) facial muscles exhibit and how a contracted facial muscle appears during clinical imaging. Such knowledge is of great clinical relevance as it will help practitioners to identify if and what type of contraction pattern is displayed by the respective muscle investigated when looking at muscle parameters like length, width, and thickness. Gaining insights into these contraction behaviors could have a wide range of clinical applications. For instance, in reconstructive medicine, this knowledge could enhance surgical and rehabilitation approaches for patients with conditions like Bell’s palsy. In aesthetic medicine, it could inform the techniques used in facelifts and the administration of neuromodulators. Understanding these contraction patterns more clearly is crucial for achieving more natural and effective outcomes in both therapeutic and cosmetic procedures. Therefore, the objective of this study is to investigate the type of muscle contraction pattern of the most prominent facial muscle—the zygomaticus major muscle (ZMa)—and its imaging-related presentation. This will be performed by utilizing MR imaging during various facial expressions.

## Materials and Methods

### Study Population

This study was designed as an observational, cross-sectional, interventional imaging-based study and was conducted in accordance with the ethical principles outlined in the declaration of Helsinki (as revised in 2013). The study design was approved by the Jagiellonian University Ethics Committee under the IRB number: NO 1072.6120.209.2022. Each participant provided written informed consent for enrollment in the study and the use of their demographic and imaging-related data.

No specific inclusion criteria were applied to allow for a community-based approach to data collection in order to represent a generally applicable dataset.

Participants were not included in this study if they had contraindications to undergo magnetic resonance imaging (metal implants) or if they had any previous injuries or procedures performed to their faces that could impair their ability to perform various facial expressions or that could impair the visibility of their facial structures during MR image analyses.

### Investigative Procedures

**Fig. 1:**
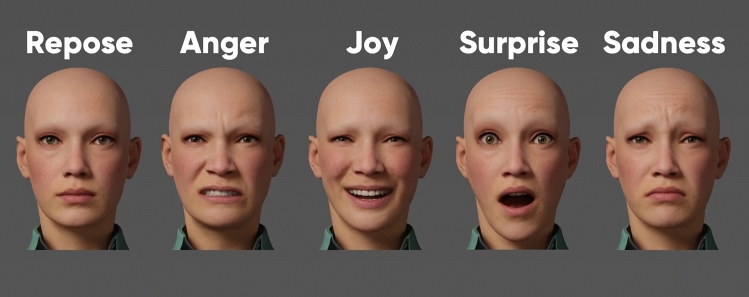
Three-dimensional (3D) facial models illustrating the five facial expressions investigated in this study: repose, anger, joy, surprise, and sadness. Participants were trained on these virtual models before their MR scanning procedure. Images were sampled from the free web portal: www.unrealengine.com

Study participants were asked to perform four facial expressions according to a facial animation chart—anger, joy, surprise, and sadness—before entering the MRI device (Fig. 1). These facial expressions were practiced in front of a mirror or front of their cell phone for purposes of consistency.

Once completed, participants were asked to enter the MRI device in the supine position and a relaxed facial expression. After the first MR scan was completed (in repose), participants were asked to perform the next facial expression (anger) and to maintain that facial position during the subsequent scan. A total of five scans were conducted—baseline/repose and all four facial expressions (anger, joy, surprise, and sadness). The duration of the MRI for each facial expression was 4 min; this was followed by a 15-s relaxation time before the next expression and scan resulting in a total scanning duration per participant of 21 min (5 × facial expressions + 4 × relaxation periods).

### MR Imaging Sequence

MR imaging data was acquired using a 1.5T Siemens Sola MR System with a 20-channel coil. A custom T1 MPRAGE sequence was performed with the following parameters: TR = 2340 ms, TE = 5.1 ms, TI = 1180 ms, FA = 8°, FOV = 240 × 240 mm, slice thickness = 0.9 mm, 288 axial slices.

### MR Image Analysis

**Fig. 2:**
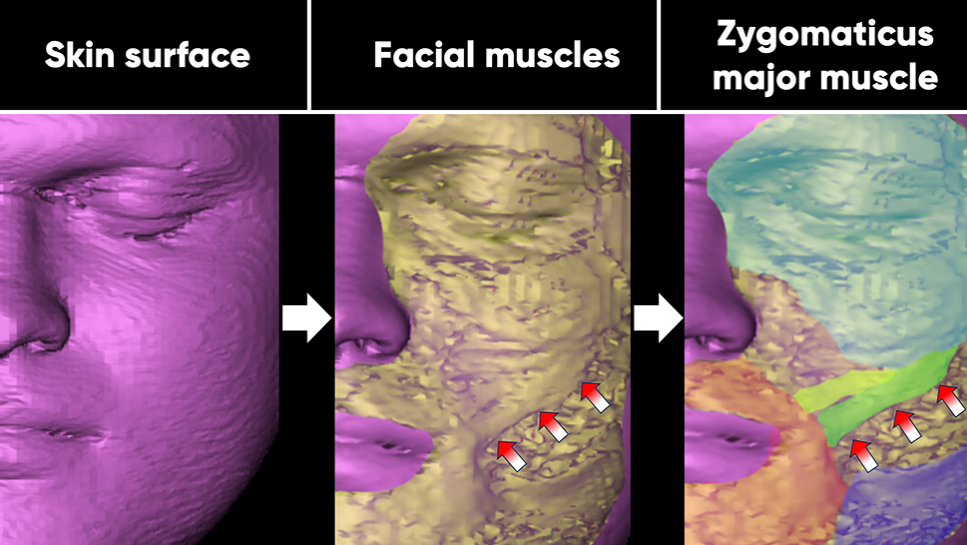
Three-dimensional (3D) facial reconstruction of one of the study participants. An extracted ZMa superimposed in the corresponding topography on the facial model B Representation of subcutaneous soft tissue structures C Color map of selected facial muscles. Red and white arrows represent the zygomaticus major muscle

**Fig. 3:**
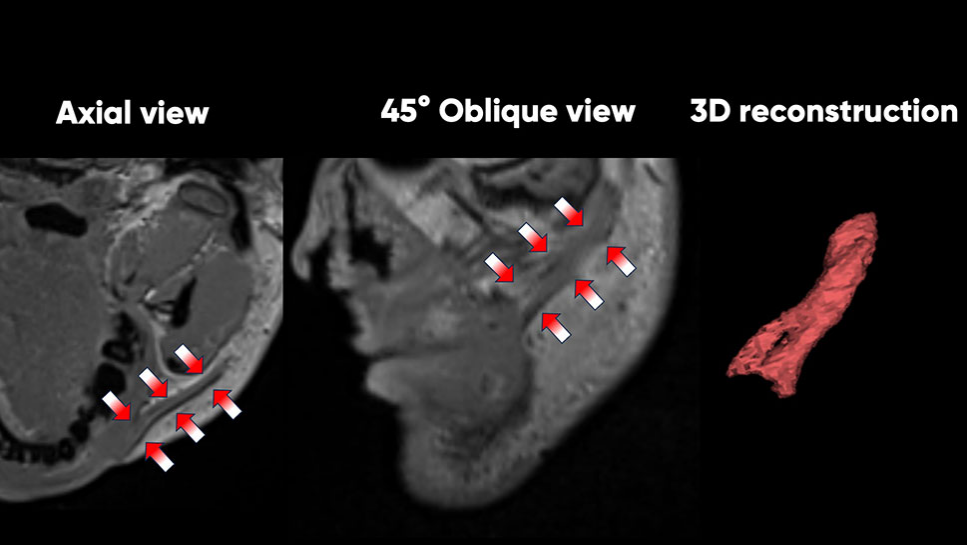
Representation of the multiplanar reconstruction from which the slice was highlighted and measured for the zygomaticus major muscle. The location and method of MRI slice acquisition are shown by the red rectangle superimposed on a facial model

Image analysis was performed using a standard radiology workstation (syngo. via software, Siemens AG, Germany). Upon image quality completion by two radiologists, MR datasets were analyzed by two operators using the multiplanar mode for image reconstruction in all three axes (Figs. 2, 3). All measurements were performed two times and the mean value was utilized for further calculations. The following measurements were manually conducted*:*

**Fig. 4:**
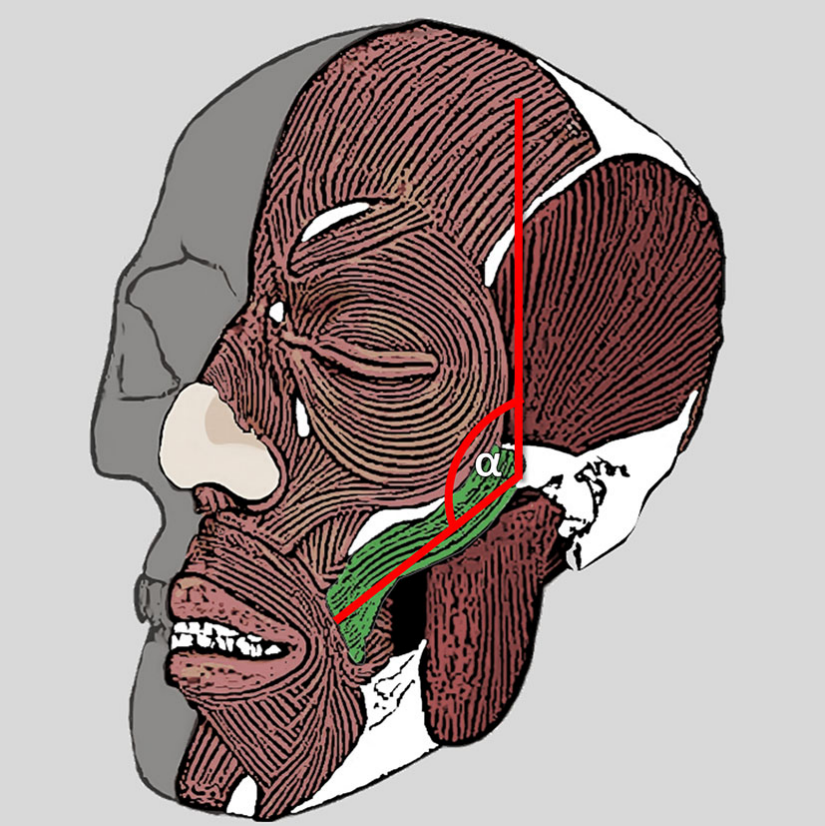
Representation of facial angle measurement methodology—the arms of the angle include the long axis of the face and the long axis of the zygomaticus major muscle

1. Length of the ZMa = Linear distance between the origin of the muscle at the zygomatic arch and its insertion at the modiolus
2. Width of the ZMa = Distance between the most cranial and most caudal aspect of the muscle at its origin, midportion, and insertion
3. Thickness of the ZMa = Distance between the most superficial and deepest aspect of the muscle at its origin, midportion, and insertion
4. Facial angle = Angle between the longitudinal axis of the ZMa and the coronal plane when measured in the (adjusted) sagittal plane starting from its origin at the zygomatic arch (Fig. 4).

### Statistical Analysis

Parameters describing the investigated muscles in their repose facial expression are given as absolute values. Parameters describing the investigated muscles during their various facial expressions, ratios were computed comparing each muscle to its own status in repose, this way each muscle served as its own control. Values greater than 100% indicate an increase, whereas values smaller than 100% indicate a decrease in that respective value. Comparisons were conducted between the five facial expressions. Comparative statistical analyses were conducted as a one-sample *t* test (test against 100%) and paired student *t* test (facial expression against repose) using SPSS 29.0 (Predictive Solutions, Pittsburgh, PA, USA). A *p* value less than 0.05 was considered statistically significant to guide conclusions.

## Results

### General Description

A total of 34 healthy young individuals of Caucasian Polish descent were investigated (17 women and 17 men) having a mean age of 23.6 (2.4) years [range: 20–30] and a mean body mass index (BMI) of 22.8 (2.3) kg/m^2^ [range: 18.6–27.8]. In repose, the average length of the ZMa was 44.77 mm (5.1) [range: 31.8–55.6] with *p *= 0.001 for gender differences.

The average width of the ZMa at its origin/midportion/insertion was 7.19 mm (2.1) [range: 3.6–13.3]/8.97 mm (2.0) [range: 4.9–14.8]/13.50 mm (6.8) [range: 2.8–32.0] with *p *= 0.013/*p *= 0.013/*p *= 0.007 for gender differences, respectively.

The average thickness of the ZMa at its origin/midportion/insertion was 3.19 mm (1.1) [range: 1.3–6.1]/3.85 mm (1.1) [range: 1.2–6.8]/3.68 mm (1.3) [range: 1.0–7.3] with *p *= 0.212/*p* < 0.001/*p *= 0.950 for gender differences, respectively.

The facial angle was in repose 136.6° (9.7) [range: 105–157] with *p *= 0.231 for gender differences (Table 1).

**Table 1:** Summary of the parameters investigated for the zygomaticus major muscle in each facial expression: repose, anger, joy, surprise, and sadness). Values are presented as mean with the respective +/− standard deviation along with the corresponding data range [min.–max. value]

	Length	Width			Thickness			Facial angle
		*Origin*	*Midportion*	*Insertion*	*Origin*	*Midportion*	*Insertion*	
Repose	44.8 (5.1) [31.8–55.6]	7.2 (2.1) [3.6–13.3]	9.0 (2.0) [4.9–14.8]	13.5 (6.8) [2.8–32.0]	3.2 (1.1) [1.3–6.1]	3.8 (1.1) [1.2–6.8]	3.7 (1.3) [1.0–7.3]	136.6 (9.7) [104.8–156.7]
Anger	44.2 (5.5) [28.0–56.3]	7.3 (2.3) [3.1–14.7]	8.7 (1.9) [4.6–13.0]	14.1 (7.2) [3.6–29.1]	2.9 (1.0) [1.0–5.2]	3.5 (0.9) [1.2–5.7]	3.3 (1.7) [1.3–13.9]	137.2 (8.6) [104.1–152.4]
Joy	38.5 (6.8) [25.2–53.6]	7.1 (2.3) [2.7–13.8]	9.1 (2.2) [5.2–15.1]	13.8 (6.7) [3.0–30.5]	3.5 (1.4) [1.1–7.8]	4.0 (1.3) [1.4–7.7]	4.1 (1.5) [1.4–8.0]	139.3 (9.1) [111.6–155.9]
Surprise	46.6 (6.1) [30.0–63.7]	7.8 (4.5) [3.0–40.2]	8.5 (1.9) [4.0–13.3]	15.2 (8.1) [3.8–47.7]	3.1 (1.1) [1.2–5.8]	3.5 (1.1) [1.6–6.3]	3.1 (1.0) [1.2–5.4]	138.5 (6.3) [116.0–152.2]
Sadness	45.7 (5.5) [33.2–59.9]	7.8 (4.1) [3.5–36.5]	8.7 (1.8) [4.6–14.4]	14.2 (7.4) [3.4–40.4]	2.9 (1.1) [1.0–6.4]	3.4 (1.1) [1.5–7.2]	3.2 (1.1) [1.3–5.7]	137.6 (8.2) [111.6–148.2]

### Facial Expression: Joy

**Fig. 5:**
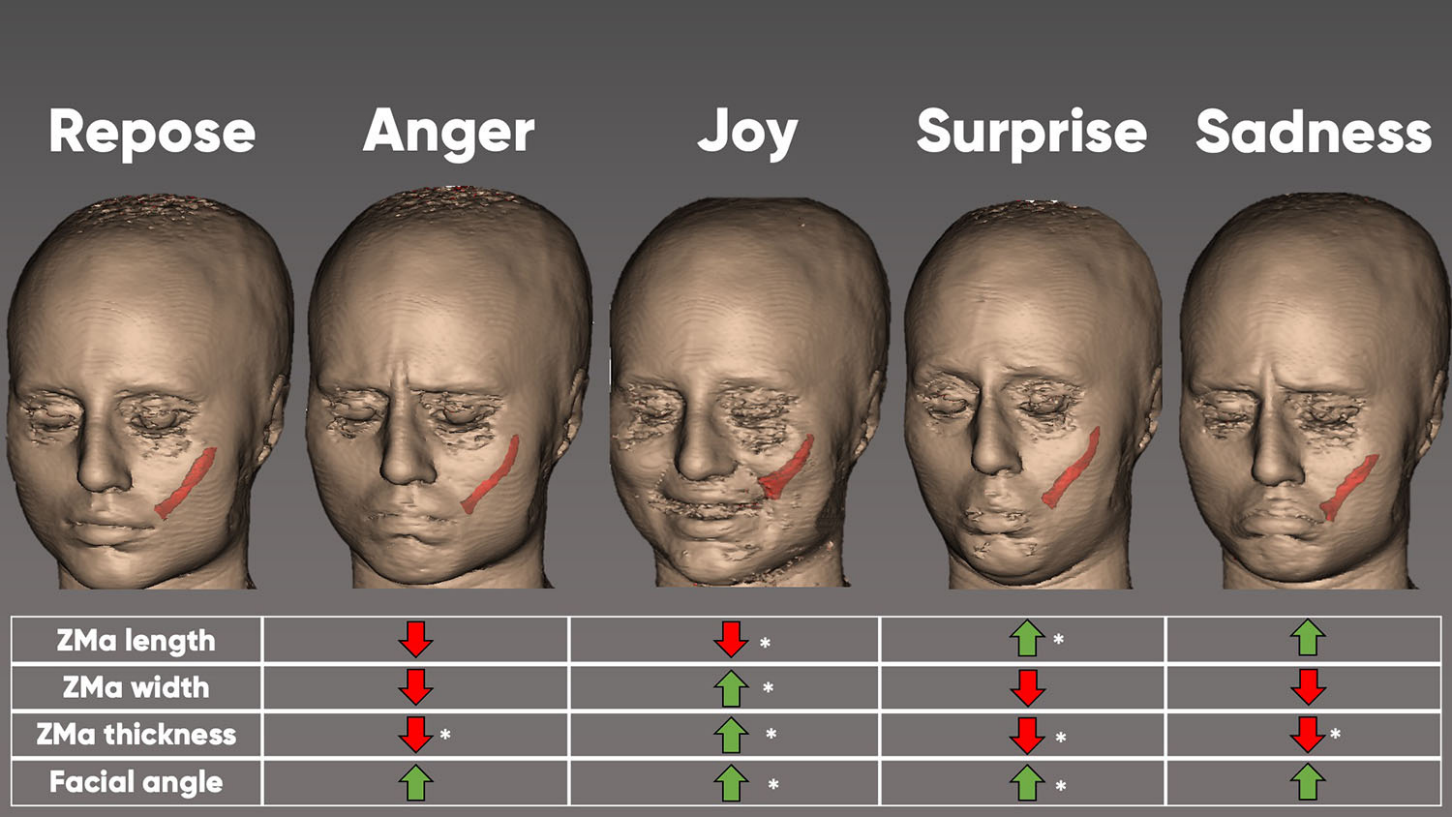
A visual comparison of the zygomaticus major muscle contractility in various expressions of the face depending on several factors—muscle length, width, thickness, and facial angle. Green arrows represent an increase, while red arrows represent a decrease in value

Upon smiling, the length of the ZMa reduced to 85.6% (11.3) from its original length (in repose 100%) (*p *< 0.001), whereas its width increased to 103.4% (21.6) (*p *= 0.195) and its thickness increased to 108.4% (19.2) (*p *= 0.001). The measured parameters are reflective of smiling, which shortens the distance between the zygomatic arch and modiolus; this is a sign of isotonic muscular contraction. This type of contraction pattern is supported by the increase in the ZMa’s thickness. The facial angle increased by 2.72° (7.8) with *p *= 0.005—a measure of the modiolus moving laterally (Fig. 5).

### Facial Expression: Anger

Upon performing an angry facial expression, the length of the ZMa was 98.9% (8.9) of its original length (*p *= 0.327), its width was 98.8% (16.2) (*p* = 0.537), and its thickness decreased to 91.15% (12.4) (*p *< 0.001). The measured parameters reflect a facial expression in which the ZMa is being passively shortened by other facial muscles that did not require the ZMa to contract. This is supported by the slight decrease in width but in the highly statistically significant reduction in thickness. The facial angle increased by 0.55° (5.4) with *p* = 0.406 following the movement of the modiolus along the longitudinal axis of the ZMa (Fig. 5).

### Facial Expression: Surprise

Upon performing a surprised facial expression, the length of the ZMa increased to 104.3% (10.7) (*p *= 0.002), its width decreased to 96.5% (21.2) (*p* = 0.175), and its thickness decreased to 91.0% (16.4) (*p* < 0.001). The measured parameters are in line with a stretching phenomenon that increases the length and decreases the width and thickness of the muscle. The facial angle increased by 1.85° (7.2) with *p* = 0.038 following the movement of the modiolus laterally to open the oral aperture (Fig. 5).

### Facial Expression: Sadness

Upon expressing sadness, the length of the ZMa was 102.7% (11.5) of its original (*p* = 0.056), its width was 99.4% (21.0) (*p* = 0.905), and its thickness decreased to 91.0% (17.4) (*p* < 0.001). The measured parameters are in line with a stretching phenomenon that increases the length and decreases the with and thickness of the muscle. The facial angle increased by 1.00° (6.7) with *p* = 0.225 representing the movement of the ZMa caudally following the contraction of the depressor anguli oris muscle (Fig. 5).

## Discussion

This MRI study investigated the contraction pattern of the ZMa as the most prominent muscle of facial expression in the midface. The study design was based on the MRI of young (age range: 20–30 years) volunteers in various facial expressions. Each person enrolled in the study had no history of facial trauma, nor any facial aesthetic procedure. The investigated facial expressions were joy, anger, surprise, and sadness as well as the repose facial position which served as the control. The conducted facial expressions were performed inside the MRI device and were maintained for 4 min by the volunteers followed by a 15-s relaxation and recovery interval. Despite the exercise of each facial expression, it can be assumed that during the scanning time, slight movements of the facial soft tissues occurred (e.g., during swallowing); this may have influenced the accuracy of the image capture and needs to be regarded as a limitation of this study. To account for such movement artifacts, images were screened by independent and experienced radiologists for quality control. Additionally, the manual measurements were performed by two operators; upon agreement between their measurements, the mean value of their outcomes was used for further calculations.

Analysis of the data revealed that the ZMa shortens in length and increases in width and thickness during smiling (Fig. 5). These measurements indicate that the ZMa undergoes a contraction pattern that is consistent with an isotonic muscle contraction in which the origin of the muscle moves closer to its insertion upon activation. However, it must be noted that the thickness of the muscle also increased significantly (*p* = 0.001); this is novel as an imaging-based observation. When compared to the other investigated facial expressions, joy/smiling was the only one to increase thickness. This finding is novel and suggests that the evaluation of muscle thickness is an important differentiating factor when it comes to identifying muscular activity. In the past, a facial expression was regarded as a sign of a muscle’s proper function during various clinical examination procedures. However, the results of this study suggest that measuring a muscle’s thickness might be more reflective of a muscle’s function and innate activity. The other conducted facial expressions are likewise capable of changing the length (anger = shortening; surprise = lengthening; sadness = lengthening) or the angle of the ZMa (all angle measurements increased), but the measurement of thickness seems to be the most reflective of a muscle’s innate activity.

Similarly, and in line with the recent results published by Calomeni et al. [10], it can be assumed that if a facial muscle is fixed to adjacent structures (e.g., bone or soft tissues with the latter due to adhesions formed by scars or implants or following facial trauma), the contraction pattern changes form isotonic to isometric. The latter contraction pattern results from the muscle’s inability to approach the point of insertion from the point of origin, thus not moving the overlying skin. This is termed isometric. Clinically, this would be assessed as a loss of function of the respective muscle because the muscle is unable to move the overlying skin. However, based on the results of the present study, the change in length and angle are insufficient parameters to evaluate proper facial muscle function. Instead, imaging methodologies should be utilized which can measure facial muscle thickness to determine if the muscle receives neural input and contracts. This contraction pattern can be either isotonic (with skin movement) or isometric (without skin movement); the latter is not assessable via facial animation.

Further, the use of EMG might be difficult due to its availability and due to its interpretability. The muscles of facial expression are arranged in layers and most muscles form muscle complexes [2, 14]. Therefore, it is difficult to obtain proper spatial resolution between the muscles and to decipher which signals belong to which muscles; this is despite recent studies that have established a protocol for most optimal measurement locations when using surface-derived EMG analyses [8, 9, 15].

The results presented herein highlight the importance of facial imaging modalities like MRI or facial ultrasound to evaluate proper facial muscle function. Facial ultrasound, in particular, has gained increased popularity during the last few years due to its ability to evaluate facial blood flow and simultaneously guide hyaluronidase injections [16–18]. The latter is of utmost importance in cases where hyaluronic acid-based soft tissue fillers have been inadvertently injected in the intravascular space; this complication can cause tissue necrosis and injection-related visual compromise [19, 20]. It could be therefore of clinical importance to implement facial ultrasound into the surgical and non-surgical routine to evaluate facial muscle activity aside from the above-mentioned indications.

However, this study is not free of limitations. First, the sample size is highly selective with only young and procedure-naïve participants included. The results might differ if another study population or another ethnic group would have been included. However, the above-mentioned items can be regarded as potential for future investigations which will expand on the results presented herein. Second, this study investigated only the ZMa despite a plethora of other facial muscles being imaged. The reason we chose to use the ZMa was due to its size and the ease of measurement methodology; future studies will need to evaluate of other facial muscles to expand on the results presented herein.

## Conclusion

The results of this MRI-based study reveal that the zygomaticus major muscle undergoes an isotonic muscular contraction especially during the facial expression of joy with a decrease in length and an increase in its width and thickness. Combining the data from the analysis of all the other facial expressions, the measurement of muscular thickness seems to be the most reliable parameter when evaluating accurate facial muscle function. Measuring muscle thickness in repose and during other facial expressions as a surrogate parameter of a muscular contraction will hopefully prove useful in different clinical situations when muscle activity needs to be evaluated. Based on the results obtained, the lack in ability to move facial soft tissues and to thus perform any facial expression is not indicative for an absent muscle function. Instead, the respective muscle thickness could be measured to truly evaluate its function. This fact can be utilized during preoperative and postoperative evaluation in reconstructive and facial transplant settings, and other circumstances where accurate functional assessment of the muscles of facial expression is necessary.
